# Association between cognitive impairment and comprehensive weight phenotypes among older adults in China: a nationwide survey

**DOI:** 10.1007/s40520-025-03068-7

**Published:** 2025-07-03

**Authors:** Yahong Gu, Yu Zhang, Xinyu Yang, Youpeng Guo, Dongyan Lu, Li Chen, Yanfang Hong

**Affiliations:** 1 Deqing People’s Hospital, 120 Yingxi South Road, Deqing County, Huzhou, Zhejiang 313200 China; 2 Department of Nursing, College of Medical Science, Huzhou University, 759 Second Ring Road, Huzhou District, Zhejiang, 313000 China; 3 College of Nursing, Hunan Normal University, No. 36, Lushan Road, Yuelu District, Changsha City, Hunan 410081 China; 4 The Second People’s Hospital of Yuhuan, No. 77, Huanbao Middle Road, chumen Town, Yuhuan City, Taizhou, Zhejiang 317605 China

**Keywords:** Older adults, Body mass index, Weight-adjusted-waist index, Cognitive impairment, Restricted cubic spline

## Abstract

**Objective:**

To investigate the relationship between obesity and cognitive impairment among older adults using body mass index (BMI) and weight-adjusted waist circumference index (WWI).

**Methods:**

Drawing on the data from the Chinese Longitudinal Healthy Longevity Survey database spanning from 2011 to 2018, the study employed Cox regression analysis and the restricted cubic spline method to examine the relationship between BMI, WWI, and cognitive impairment.

**Results:**

A total of 2546 subjects were included in this study. According to BMI, lean was a risk factor for cognitive impairment in older adults (HR = 1.347, 95%CI:1.011 ~ 1.795), overweight (HR = 0.446, 95%CI:0.35 ~ 0.568) and obesity (HR = 0.225, 95%CI:0.161 ~ 0.314) was a protective factor for cognitive impairment in the older adults. According to the classification of WWI, high WWI was a risk factor for cognitive impairment in older adults (HR = 2.064, 95%CI:1.731 ~ 2.461). The restricted cubic spline showed that when WWI > 11 cm/$\sqrt {kg}$, the risk of cognitive impairment increased with the increase of WWI. The comprehensive body weight phenotype constructed by BMI and WWI showed that the older adults with lean combined with high WWI were found to have the highest risk of cognitive impairment (HR = 1.542, 95%CI:1.087–2.187), and obesity combined with low WWI had the largest protective effect on cognitive function in the older adults by(HR = 0.108, 95%CI: 0.062 ~ 0.186).

**Conclusion:**

Maintaining an appropriate level of overweight or even a state of obesity can contribute to the preservation of cognitive health in older adults. This factor holds significant importance as it is a preventive measure against the onset of cognitive impairment.

## Introduction

As the global population ages, the number of older adults with cognitive impairment is increasing; Cognitive impairment is a transitional state between normal aging and dementia, characterized by declines in memory, attention, and cognitive functioning [1].In 2015, approximately 46.8 million people were diagnosed with dementia worldwide, and this number is expected to rise to 130 million by 2050 [2, 3]. Since there are currently no targeted drug treatments for most types of dementia, such as Alzheimer’s disease, to prevent the development of dementia, attention should be focused on cognitive impairment as a transitional state between aging and dementia [4].

Previous studies have found that obesity, an emerging global problem, not only increases the risk of cardiovascular disease but also adversely affects the central nervous system and cognitive function [5, 6]. Therefore research on the relationship between obesity and cognitive health in older adults has received increasing attention. Obesity-related body measurements, such as Body mass index (BMI), are associated with certain cognitive domains [7, 8], but there are discrepancies in the findings. Some studies have linked obesity to cognitive impairment in older adults [9], while others have suggested that obesity does not increase the risk of dementia and may even reduce the risk of cognitive impairment [7, 10]. These differences may be the main indicator of the degree of obesity [11] and an over-reliance on BMI, which fails to distinguish adipose tissue from lean body mass and cannot differentiate between overall adiposity and abdominal fat distribution, potentially leading to misclassification and confounding of results [12].

Given these limitations, there is an urgent need for a more precise metric to distinguish the nuances of obesity and its potential cognitive impact. The Weight-adjusted waist index (WWI) combines waist circumference and body weight to provide a fine-grained measure of body fat distribution [13]. Kim’s research [14] has identified the Weight-adjusted Waist Index (WWI) as an anthropometric measure that correlates positively with adiposity and negatively with muscle mass in elderly individuals. Wang Shanshan [15] expanded on this by comparing the differential associations of six obesity indices with dementia in older adults, concluding that WWI exhibits a linear correlation with the risk of dementia and Alzheimer’s disease (AD). An elevated WWI may be indicative of dementia syndrome and Alzheimer’s dementia, serving as a potential clinical marker. Current studies often rely on BMI, which fails to distinguish between fat distribution and muscle mass. WWI, by integrating waist circumference and body weight, offers a more sensitive reflection of central obesity and body composition ratios. The concurrent use of BMI (for overall obesity) and WWI (for fat distribution) enables a more holistic evaluation of the relationship between obesity phenotype and cognition, mitigating classification bias. This integrated approach to assessing weight phenotype, combining BMI and WWI, is essential for clarifying the nexus between obesity and cognitive function. A comprehensive weight phenotype that unites BMI and WWI may be crucial for elucidating the link between obesity and cognition.

Therefore, the present study proposed to explore the association between BMI, WWI, and the two jointly with cognitive impairment in old age through Cox regression and restricted cubic spline (RCS) modeling based on data from the Chinese Longitudinal Health and Longevity Survey (CLHLS) to establish a more robust evidence-based foundation for formulating intervention protocols targeting cognitive impairment.

## Methods

### Data sources and study population

The research dataset was derived from the CLHLS, encompassing a nationally representative sample of 500 older adult participants across diverse age groups from 23 provincial-level administrative divisions throughout China. This study has applied for and obtained the right to use the data through the Peking University Open Research Data Platform. CLHLS received ethical approval from the Biomedical Ethics Committee of Peking University in China (IRB00001052-13074).

This study selected older adult people who completed the three surveys in 2011, 2014, and 2018 respectively, were ≥ 60 years old, excluding samples with missing key variables, and finally included 2546 individual samples of older adult people.

### Research methods

#### Measurement of cognitive function

Cognitive function assessment was conducted using the standardized Chinese adaptation of the Mini-Mental State Examination (MMSE), a 24-item instrument with a scoring range from 0 to 30, where decreasing scores indicate progressively impaired cognitive performance. The boundaries were set according to the education level of the subjects, and cognitive impairment was defined when the middle school and above group scored ≤ 24, the elementary school group scored ≤ 20, and the illiterate group scored ≤ 17 [16]. 

#### Obese assessment

BMI was calculated using anthropometric measurements of height and weight obtained from the survey, applying the standard formula: BMI = weight (kg) / height (m)². Based on established classification criteria, participants were categorized into four groups: lean (BMI < 18.5 kg/m²), normal weight (18.5 ≤ BMI < 23.9 kg/m²), overweight (24 ≤ BMI < 28 kg/m²), and obese (BMI ≥ 28 kg/m²) [17]. The WWI was computed based on anthropometric measurements of body weight and abdominal circumference obtained through the survey instrument, and the formula was: waist circumference (cm)/$\:\surd\:\text{w}\text{e}\text{i}\text{g}\text{h}\text{t}\left(\text{k}\text{g}\right)$, when WWI < 11.25 cm/$\sqrt {kg}$ was defined as low WWI, and WWI ≥ 11.25 cm/$\sqrt {kg}$ was defined as high WWI [18]. In addition, a comprehensive weight status classification was constructed in this study based on weight classifications divided by BMI and WWI.

#### Covariates

General demographic characteristics include age, gender, place of residence, education, marital status, and older adults’ care situation. Lifestyle factors include smoking, alcohol consumption, and exercise. Other variables include sleep quality, incapacity, and multimorbidity. Disability was assessed using a scale developed by Katz [19]; multimorbidity was defined as the simultaneous presence of two or more chronic diseases [20].

### Statistical methods

Statistical analyses were conducted utilizing the R statistical package (version 4.3.3) and SPSS software (version 25.0). Categorical variables were expressed as frequencies and proportions, while intergroup comparisons of baseline characteristics were performed using Pearson’s chi-square test. We used the Cox proportional hazards model to analyze the association between BMI, WWI, and cognitive impairment and adjusted for multiple covariates. To further assess the dose-response relationship among BMI, WWI, and muscle mass, we construct an RCS (Restricted Cubic Splines) model and select the optimal RCS model based on the criterion of minimizing the Akaike Information Criterion (*AIC*). The reference values were automatically determined by the RCS algorithm as the BMI and WWI values associated with the lowest risk of cognitive impairment (*HR* = 1) in the study population, identified through smoothed spline fitting and minimization of the *AIC*. In addition, the correlation between a comprehensive weight phenotype and cognitive impairment in older adults was examined by subgroup analysis. To ensure the robustness of our research findings, we conducted a sensitivity analysis by adjusting the classification thresholds for BMI and WWI, and the significance level adopted for the statistical tests was set at 0.05.

## Results

### Sample descriptive analysis

Finally, 2546 older adults were included as study participants. After 7 years of follow-up, there were 571 (22.46%) new cases of cognitive impairment, with an incidence density of 3.21/100 person-years; the differences in age, gender, place of residence, education, marital status, sleep quality, incapacity, multimorbidity, eldercare situation, smoking, BMI and WWI were statistically significant between the two groups (all *P* < 0.001), and the specific data are detailed in Table 1.

**Table 1:** Baseline characteristics of the study population(*n* = 2546)

Characteristics	Cognitive impairment ($\bar {x}\pm{s}$ / %)		x^2^	*P*
	No	Yes		
Age (year)			79.156	< 0.001
60~	392(19.85)	40(7.01)		
70~	948(48)	252(44.13)		
80~	471(23.85)	203(35.55)		
90~	164(8.3)	76(13.31)		
Gender			78.266	< 0.001
Male	1093(55.34)	196(34.33)		
Female	882(44.66)	375(65.67)		
Place of residence			8.291	< 0.001
City	291(14.73)	63(11.03)		
Towns	571(28.91)	194(33.98)		
Countryside	1113(56.35)	314(54.99)		
Education			183.353	< 0.001
Illiterate	804(40.71)	385(67.43)		
Primary school education	811(41.06)	155(27.15)		
Junior high school education	320(16.2)	30(5.25)		
High school education and above	40(2.03)	1(0.18)		
Marital status			55.113	< 0.001
Married	1237(62.63)	260(45.53)		
Unmarried	27(1.37)	7(1.23)		
Divorce/Widowed	711(36)	304(53.24)		
Sleep quality			28.045	< 0.001
Good	1298(65.72)	322(56.39)		
Normal	477(24.15)	148(25.92)		
Bad	200(10.13)	101(17.69)		
incapacity			50.239	< 0.001
Yes	55(2.78)	55(9.63)		
No	1920(97.22)	516(90.37)		
Multimorbidity			7.710	< 0.001
Yes	572(28.96)	200(35.03)		
No	1403(71.04)	371(64.97)		
Elderly care situation			11.348	< 0.001
With family	1615(81.77)	431(75.48)		
Alone	343(17.37)	132(23.12)		
Elderly care institutions	17(0.86)	8(1.4)		
Smoke			8.262	< 0.001
Yes	446(22.58)	97(16.99)		
No	1529(77.42)	474(83.01)		
Alcohol consumption			3.384	0.066
Yes	430(21.77)	104(18.21)		
No	1545(78.23)	467(81.79)		
Exercise				
Yes	795(40.25)	221(38.7)		
No	1180(59.75)	350(61.3)		
WWI			116.934	< 0.001
High	1271(64.35)	223(39.05)		
Low	704(35.65)	348(60.95)		
BMI			168.218	< 0.001
Normal	808(40.91)	345(60.42)		
Lean	157(7.95)	101(17.69)		
Overweight	527(26.68)	82(14.36)		
Obesity	483(24.46)	43(7.53)		

### BMI subgroups and risk of cognitive impairment in older adults

**Fig. 1:**
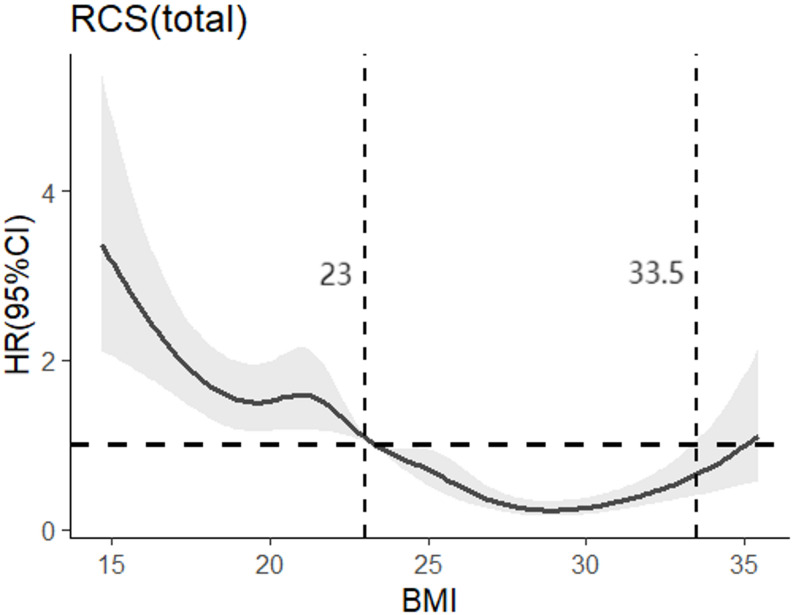
Dose-response relationship between BMI and cognitive dysfunction in older adults

With the presence of cognitive impairment as the dependent variable, and BMI as the independent variable. After adjusting for all confounders, wasting was a risk factor for cognitive impairment in older adults (*HR* = 1.347, 95%*CI*:1.011 ~ 1.795), and overweight (*HR* = 0.446, 95%*CI*:0.35 ~ 0.568) and obesity (*HR* = 0.225, 95%*CI*:0.161 ~ 0.314) were protective factors for cognitive impairment in older adults, as shown in Table 2. The relationship between BMI and cognitive impairment was further analyzed by applying the RCS model, and according to the spline regression coefficient and the *AIC* criterion, the present study found that *AIC* = 8598.542 reached the minimum among the relevant models with 7 nodes. The results showed a nonlinear dose-response relationship between BMI and the risk of developing cognitive impairment in older adults (*P*_*total trend*_ <0.05, *P*_*nonlinear*_ <0.05). The risk of prevalence of cognitive impairment increased sharply with decreasing BMI when BMI was < 23 kg/m^2^. The relationship between the risk of incidence of cognitive impairment and BMI was not significant when BMI was > 33.5 kg/m^2^. They are shown in Fig. 1.

**Table 2:** Cox regression results for BMI subgroups and cognitive impairment in older adults

BMI	Mould 1[HR(95%CI)]	Mould 2[HR(95%CI)]	Mould 3[HR(95%CI)]
Normal	Reference group	Reference group	Reference group
Lean	1.507(1.139 ~ 1.993)	1.436(1.15 ~ 1.792)	1.347(1.011 ~ 1.795)
Overweight	0.422(0.332 ~ 0.537)	0.435(0.342 ~ 0.554)	0.446(0.35 ~ 0.568)
Obesity	0.252(0.183 ~ 0.346)	0.186(0.135 ~ 0.257)	0.225(0.161 ~ 0.314)

Note: Mould 1 is a one-factor model; Mould 2 adjusts for age and gender on the basis of Mould 1; Mould 3 adjusts for place of residence, education, marriage, sleep quality, disability, multimorbidity, pension status, smoking, alcohol consumption, and exercise on the basis of Mould 2

### WWI subgroups and risk of cognitive impairment in older adults

**Fig. 2:**
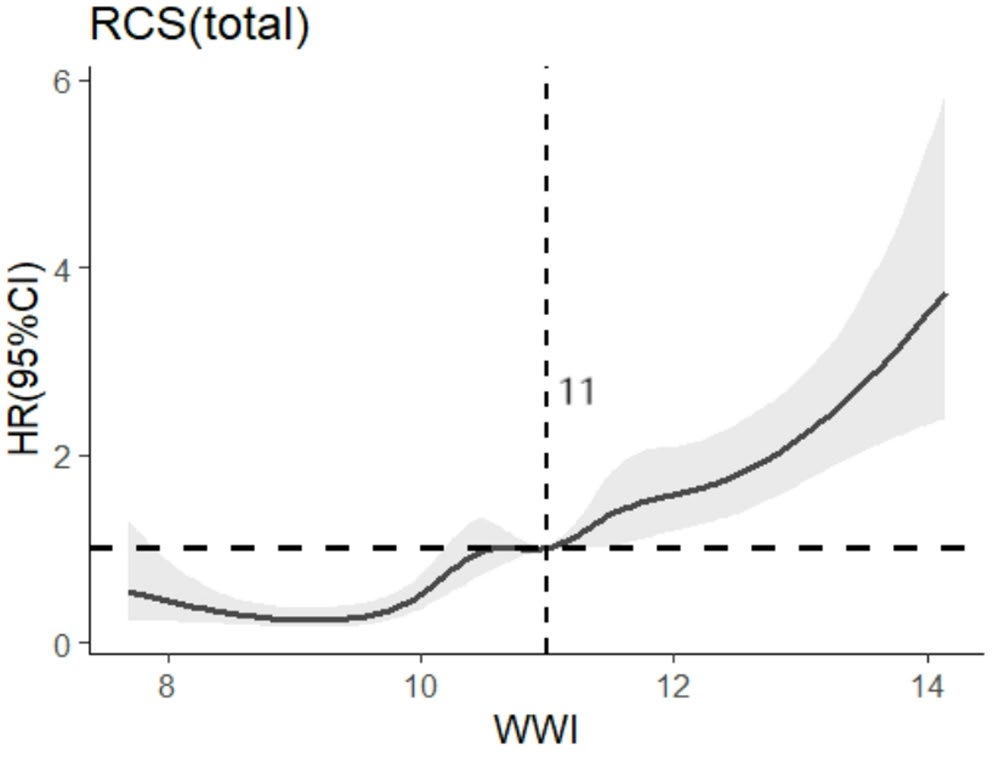
Dose-response relationship between WWI and cognitive dysfunction in older adults

With the presence of cognitive impairment as the dependent variable and WWI as the independent variable, high WWI was a risk factor for cognitive impairment in older adults after adjusting for all confounders (*HR* = 2.064, 95%*CI*:1.731 ~ 2.461), as shown in Table 3. The relationship between WWI and cognitive impairment was further analyzed by applying the RCS model, and according to the spline regression coefficients and the *AIC* criterion, this study found that *AIC* = 8634.145 reached the minimum among the relevant models with 7 nodes. The modeling results showed a nonlinear dose-response relationship between WWI and the risk of developing cognitive impairment in older adults (*P*_total trend_ < 0.05, *P*_nonlinear_ < 0.05). The risk of incidence of cognitive impairment increased with increasing WWI when WWI > 11 cm/$\sqrt {kg}$. See Fig. 2 for details.

**Table 3:** Cox regression results of WWI subgroups and cognitive impairment in older adults

WWI	Mould 1[HR(95%CI)]	Mould 2[HR(95%CI)]	Mould 3[HR(95%CI)]
Low	Reference group	Reference group	Reference group
High	2.367(2.001 ~ 2.801)	2.136(1.798 ~ 2.537)	2.064(1.731 ~ 2.461)

Note: See Table 2

### BMI + WWI composite defined composite weight phenotype and risk of cognitive impairment in older adults

With the presence of cognitive impairment as the dependent variable, the BMI + WWI composite defining subgroup as the independent variable, and normal combined with low WWI as the reference group. After adjusting for all confounders, normal combined with high WWI (*HR* = 1.524, 95%*CI*:1.218 ~ 1.906), lean combined with low WWI (*HR* = 1.533, 95%*CI*:1.11 ~ 2.118), and lean combined with high WWI (*HR* = 1.542, 95%*CI*:1.087 ~ 2.187), are risk factors for cognitive impairment in older adults individuals. Overweight combined with low WWI(*HR* = 0.423, 95%*CI*:0.29 ~ 0.619), overweight combined with high WWI(*HR* = 0.703,95%*CI*:0.501 ~ 0.986), and obesity combined with low WWI(*HR* = 0.108, 95%*CI*:0.062 ~ 0.186) are protective factors for cognitive impairment in the older adults. Once the confounders were taken into account, the combination of obesity and a high WWI did not show a significant correlation with the risk of cognitive impairment among older adult individuals (*HR* = 0.719, 95%*CI*:0.473 ~ 1.093). See Table 4 for details.

**Table 4:** Cox regression results of combined weight phenotype and cognitive impairment in older adults

Combined weight phenotype	Mould 1[HR(95%CI)]	Mould 2[HR(95%CI)]	Mould 3[HR(95%CI)]
Normal/Low WWI	Reference group	Reference group	Reference group
Normal/High WWI	1.998(1.607 ~ 2.483)	1.548(1.239 ~ 1.934)	1.524(1.218 ~ 1.906)
Lean/Low WWI	1.76(1.247 ~ 2.483)	1.574(1.114 ~ 2.224)	1.533(1.11 ~ 2.118)
Lean/High WWI	2.407(1.767 ~ 3.28)	1.607(1.171 ~ 2.206)	1.542(1.087 ~ 2.187)
Overweight/Low WWI	0.418(0.287 ~ 0.61)	0.425(0.291 ~ 0.619)	0.423(0.29 ~ 0.619)
Overweight/High WWI	0.907(0.652 ~ 1.263)	0.716(0.512 ~ 1.001)	0.703(0.501 ~ 0.986)
Obesity/Low WWI	0.17(0.1 ~ 0.29)	0.108(0.063 ~ 0.185)	0.108(0.062 ~ 0.186)
Obesity/High WWI	0.939(0.624 ~ 1.412)	0.722(0.477 ~ 1.092)	0.719(0.473 ~ 1.093)

Note: See Table 2 (BMI and WWI do not have multicollinearity, Variance Inflation Factor (*VIF*) = 1.022.)

### Subgroup analysis

**Fig. 3:**
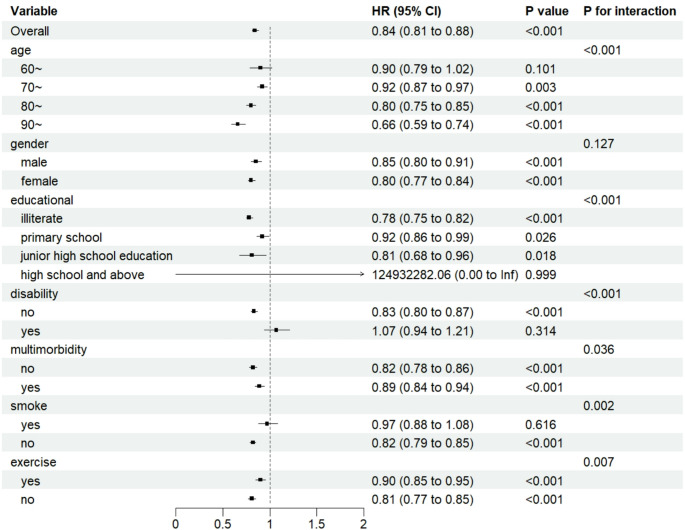
Subgroup analysis of the association between combined weight phenotype and cognitive dysfunction in older adults

Subgroup analyses were used to examine the relationship between a comprehensive weight phenotype and cognitive impairment in older adults in different population subgroups, stratified by age, gender, education, disability, multimorbidity, smoking status, and exercise, and the results are presented in Fig. 3. The results of the study showed that in some subgroups, the decrease in muscle mass was consistent with the emergence of incapacitation in older adults. Interaction tests showed that the relationship between muscle mass and older adults’ disability differed significantly between strata of age, education, disability, multimorbidity, smoking status, and exercise (*P*_for interaction_ <0.05) (See Table 5, 6 and 7).

**Table 5:** Cox regression results for BMI subgroups and cognitive impairment in older adults(Sensitivity analyses)

BMI	Mould 1[HR(95%CI)]	Mould 2[HR(95%CI)]	Mould 3[HR(95%CI)]
Q1	Reference group	Reference group	Reference group
Q2	0.805(0.665 ~ 0.976)	0.847(0.699 ~ 1.027)	0.874(0.720 ~ 1.060)
Q3	0.400(0.316 ~ 0.506)	0.439(0.347 ~ 0.556)	0.456(0.359 ~ 0.578)
Q4	0.193(0.142 ~ 0.262)	0.160(0.117 ~ 0.218)	0.171(0.125 ~ 0.234)

Note: See Table 2

**Table 6:** Cox regression results of WWI subgroups and cognitive impairment in older adults(Sensitivity analyses)

WWI	Mould 1[HR(95%CI)]	Mould 2[HR(95%CI)]	Mould 3[HR(95%CI)]
Low	Reference group	Reference group	Reference group
High	2.499(2.087 ~ 2.993)	2.411(2.009 ~ 2.894)	2.380(1.977 ~ 2.866)

Note: See Table 2

**Table 7:** Cox regression results of combined weight phenotype and cognitive impairment in older adults(Sensitivity analyses)

Combined weight phenotype	Mould 1[HR(95%CI)]	Mould 2[HR(95%CI)]	Mould 3[HR(95%CI)]
BMI Q1/Low WWI	Reference group	Reference group	Reference group
BMI Q1/High WWI	1.625(1.226 ~ 2.153)	1.272(0.957 ~ 1.69)	1.276(0.96 ~ 1.697)
BMI Q2/Low WWI	0.713(0.499 ~ 1.018)	0.702(0.491 ~ 1.003)	0.733(0.512 ~ 1.049)
BMI Q2/High WWI	1.383(1.037 ~ 1.843)	1.186(0.888 ~ 1.585)	1.228(0.918 ~ 1.644)
BMI Q3/Low WWI	0.333(0.218 ~ 0.509)	0.356(0.233 ~ 0.544)	0.359(0.235 ~ 0.55)
BMI Q3/High WWI	0.758(0.544 ~ 1.056)	0.645(0.462 ~ 0.901)	0.701(0.5 ~ 0.983)
BMI Q4/Low WWI	0.102(0.056 ~ 0.184)	0.069(0.038 ~ 0.125)	0.074(0.04 ~ 0.134)
BMI Q4/High WWI	0.589(0.395 ~ 0.877)	0.483(0.323 ~ 0.722)	0.506(0.338 ~ 0.759)

Note: See Table 2(BMI and WWI do not have multicollinearity, Variance Inflation Factor (*VIF*) = 1.036.)

### Sensitivity analyses

This study further validated the robustness of its results by performing sensitivity analyses with modified thresholds for BMI and WWI. BMI was categorized into four quartiles: Q1 (< 20.4 kg/m^2^), Q2 (20.4 to 23.3 kg/m^2^), Q3 (23.4 to 26.9 kg/m^2^), and Q4 (≥ 27 kg/m^2^); WWI was divided into two groups based on the mean value: low WWI (< 10.94 cm/$\sqrt {kg}$) and high WWI (≥ 10.94 cm/$\sqrt {kg}$).The results show that the outcomes of all sensitivity analyses are essentially consistent with the main analysis results, indicating that the study results are robust. Due to space limitations, these results are not displayed in the text. For details, please refer to the attachment.

## Discussion

This longitudinal study found an association between body mass index and weight-adjusted waist index and cognitive dysfunction in older adults. Analysing of the study data also noted that older adults with wasting combined with high WWI had the highest risk of developing cognitive dysfunction, the combination of obesity and lower WWI values demonstrated the most significant protective association with cognitive performance in the older adults.

In the present study, 2546 older adults were accrued and followed up, and there were 571 (22.46%) new cases of cognitive impairment, the observed incidence density reached 3.21 cases per 100 person-years, representing a statistically significant elevation compared to the previously reported rate of 2.70 cases per 100 person-years in the study conducted by Xingping Zhang et al. [21]. The reason may be that Xingping Zhang et al.‘s definition of age-related cognitive impairment differs from the evaluation method used in the present study. Research indicates that maintaining a body mass index between 23 and 33.5 kg/m² could potentially serve as a protective element against cognitive impairment in older adults, which is consistent with the results of a cohort study reported in The Lancet [22]. It is recommended that older adults should maintain high BMI levels later in life. The reason for this analysis may be that individuals with high BMI have greater nutritional reserves that help maintain basic physiological functions and can be used as a source of energy during disease or malnutrition, thus preserving cognitive function [23]. Secondly, higher BMI is associated with lower rates of bone loss [24], which not only reduces the negative impact of chronic disease on cognitive function but also mitigates the risk of falls and traumatic episodes, this protective effect supports the preservation of functional capacity, and community engagement in older adults individuals, thereby reducing the risk of cognitive impairment. It is also worth noting that cognitive impairment may lead to a decrease in appetite or a weakening of self-care abilities, which in turn can cause weight loss and a reduction in BMI, representing a form of reverse causality. In contrast, MA [25]. Identified higher levels of BMI as a potential risk factor for cognitive impairment, which is inconsistent with the results of this study. The discrepancy in these results may be due to the inherent shortcomings of BMI measurement, which provides a broad indicator of body weight, but it is difficult to differentiate between fat and muscle mass [26], and therefore it is prone to contradictions by simply going through BMI and exploring its relationship with cognitive function.

To delve into the association between obesity and cognitive function more precisely, WWI [14], which can reflect fat content and muscle mass, was chosen in this study to further explore the relationship between WWI and cognitive impairment. In this study, Research has demonstrated that waist-to-weight index (WWI) serves as a significant predictor for cognitive impairment in older adults, with a notable escalation in cognitive impairment prevalence observed at WWI values exceeding 11 cm/$\sqrt {kg}$.The findings of the current research support QIU’s discovery [11] that a higher WWI hurts the learning, memory, verbal fluency, and processing speed of older adult individuals in the United States. Previous studies have shown that WWI is an important predictor of cardiovascular morbidity and mortality [13], therefore higher WWI is associated with a variety of cardiovascular risk factors including hypertension, dyslipidemia, and atherosclerosis, which lead to reduced blood flow and compromised vascular health in the brain, and impaired cerebral blood flow and vascular impairment are associated with cognitive impairment [27].

Since measuring BMI and WWI alone may not assess the body shape of all individuals, a combined BMI + WWI phenotypic classification was also constructed in this study. This study found that older adults with lean combined with high WWI had the highest risk of cognitive impairment. The reason for this analysis may be that lean in older adults is often accompanied by malnutrition, sarcopenia, and various chronic diseases. Moreover, there exists an inverse correlation between WWI and bone as well as muscle mass. Conversely, WWI shows a positive correlation with fat mass [28], this situation has the potential to augment the burdens on the cardiovascular and cerebrovascular systems, thus affecting the cognitive health of older adults. In this study, we found that obesity combined with low WWI had the greatest protective effect on cognitive function in older adults. This is consistent with the “obesity paradox” observed by DONINI [29] in older adults, suggesting that obesity in older adults may have a protective effect on their health. Therefore, this study suggests that maintaining a slightly higher BMI within an appropriate range, while avoiding the risks associated with obesity such as cardiovascular and metabolic diseases, has a positive significance for the cognitive function of middle-aged and elderly individuals. Although BMI correlates with cognitive protection, it does not distinguish between fat and muscle mass [6]. Research indicates that visceral fat, as indicated by WWI, is closely associated with chronic low-grade inflammation, insulin resistance, and lipid metabolism disorders [13, 26]. These pathological processes might hasten cognitive decline through mechanisms such as blood-brain barrier damage and neuronal oxidative stress [30, 31]. In contrast, muscle mass can enhance synaptic plasticity and neurogenesis by secreting myokines, thereby providing protection independently of fat mass [32]. Consequently, a high BMI combined with low WWI (indicating high muscle mass and low visceral fat) may offer the best cognitive protection through a dual mechanism of nutritional reserves and neuroprotection. Conversely, a low BMI with high WWI (suggesting muscle loss and visceral fat accumulation) poses the greatest risk due to metabolic disorders and neuroinflammation.

A key advantage of our research lies in employing a meticulously designed, extensive, and nationally representative prospective cohort study focusing on adults aged 65 and above in China. Also, according to our findings, this study represents a pioneering investigation in which the combined assessment of WWI and BMI, along with their composite weight phenotypes, was employed to examine the association between obesity patterns and cognitive performance among older adults Chinese. As a novel anthropometric indicator reflecting the components of fat and muscle mass, WWI can combine the advantages of waist circumference and weaken the correlation with BMI. Compared with other scholars who have used WWI or BMI indicators alone to measure individuals’ body fat levels, this composite weight phenotype is more responsive to the body shape of all individuals. Our study also has limitations, this study is based on a large database, and the comprehensive weight phenotype, which is computed based on weight, waist circumference, and height, fails to precisely mirror the body fat distribution in older adults. Second, some confounders were based on self-reported data, such as sleep quality, education, and eldercare situations, which could lead to recall bias. Furthermore, in our study, the absence of comprehensive socioeconomic data hinders our ability to precisely assess how socioeconomic status influences the relationship between obesity patterns and cognitive function. Variations in socioeconomic status may lead to substantial disparities in individuals’ dietary habits, lifestyle choices, and healthcare access, which could significantly impact both obesity and cognitive function. Consequently, these disparities introduce a potential confounding element that might distort our research findings. Thus, higher quality studies are needed in the future to further employ precision measuring instruments to accurately respond to the body fat distribution of older adults, and to deeply investigate the biological mechanisms and the cause-and-effect relationships existing between obesity and cognitive impairment.

## Conclusion

Utilizing a longitudinal study design, this research uncovered a robust association between the weight level of older adults and their cognitive impairment, introducing a new phenotype of BMI combined with WWI that more accurately describes an individual’s body shape. The results of this study have important implications for the protection of cognitive function in older adults, suggesting that excessive weight loss may not be necessary and that certain levels of body weight above the normal range may contribute to maintaining their cognitive health. While moderate overweight may have benefits, we should also be vigilant about the other health risks associated with obesity and advocate for personalized health management strategies. Therefore, this finding is important for the prevention of cognitive impairment and subsequent dementia.

## Data Availability

The data that support the findings of this study are openly available in CLHLS at https://opendata.pku.edu.cn/dataverse/CHADS, Reference No. IRB00001052-13074.
